# The Involvement of Central Noradrenergic Pathway in the Analgesic Effect of Bee Venom Acupuncture on Vincristine-Induced Peripheral Neuropathy in Rats

**DOI:** 10.3390/toxins12120775

**Published:** 2020-12-06

**Authors:** Daxian Li, Geehoon Chung, Sun Kwang Kim

**Affiliations:** 1 Department of Science in Korean Medicine, Graduate School, Kyung Hee University, Seoul 02447, Korea; lidaxian721@naver.com; 2 Department of Physiology, College of Korean Medicine, Kyung Hee University, Seoul 02447, Korea; geehoon.chung@khu.ac.kr; 3 Department of East-West Medicine, Graduate School, Kyung Hee University, Seoul 02447, Korea

**Keywords:** chemotherapy-induced peripheral neuropathy, vincristine, bee venom acupuncture, spinal wide dynamic range neuron, locus coeruleus, adrenergic receptor

## Abstract

Vincristine is a vinca alkaloid anti-mitotic drug with a broad spectrum of effects on solid and hematologic cancers. The major dose-limiting factor of this anti-cancer regimen is painful peripheral neuropathy. However, no gold-standard analgesic option has been used clinically. In this study, we investigated the effects and mechanism of bee venom acupuncture (BVA) to alleviate peripheral neuropathic pain induced by repeated intraperitoneal infusions of vincristine (1 mg/kg/day, days 1–5 and 8–12) in rats. Subcutaneous injection with bee venom (BV, 1.0 mg/kg) at the ST36 acupoint ameliorated cold and mechanical hypersensitivity (i.e., aberrant withdrawal responses in acetone drop and von Frey hair tests, respectively). In vivo extracellular recording demonstrated that BVA inhibited cutaneous cold (acetone) and mechanical (brush, press, and pinch) stimuli-elicited abnormal hyperexcitation of the spinal wide dynamic range (WDR) neurons in vincristine-treated rats. In addition, the microinjection of lidocaine into the ipsilateral locus coeruleus or the antagonism of the spinal α_2_-adrenergic receptors clearly reversed the effects of BVA on cold and mechanical hypersensitivity, indicating a vital role of the descending noradrenergic modulation in analgesia. These findings suggest that BVA could be a potential therapeutic option for vincristine-induced peripheral neuropathy.

## 1. Introduction

Vincristine, a derivative of Madagascar *Catharanthus roseus* (a species of flowering plant in the *Apocynaceae* family), is a core antineoplastic agent used to control the proliferation of lymphomas, acute lymphoblastic leukemia, and sarcomas [1]. Nonetheless, several articles have described vincristine-induced paresthesias, ongoing or evoked painful abnormalities (i.e., allodynia and hyperalgesia) in the extremities [2,3,4], which cause serious concern as an increasing number of patients received this life-saving regimen. In clinical settings, most cancer survivors experience progressive chemotherapy-induced peripheral neuropathy (CIPN) in the early stages of therapy or even years after the end of vincristine therapy [5]. Once this dose-limiting complication develops, there is a high likelihood of dose reduction or the termination of anti-cancer therapy [6]. Unfortunately, no gold-standard curative or prophylactic pharmaceutical strategy is currently available.

In conventional medicine, natural compounds isolated from animals and plants represent a rich source of analgesics [7]. Among these, bee venom (BV), a venomous mixture secreted from *Apis mellifera*, has been commonly used for pain relief, mostly by injecting it into acupuncture points (i.e., *apipuncture*) [8,9]. As several components of BV (e.g., peptide 401 and histamine) have allergic properties, such therapy could need allergy tests prior to the clinical applications [9,10]. On the other hand, articles have reported that BV suppresses allergic reactions by modulation of regulatory T cells [11]. To date, the detailed analgesic features of acupuncture with purified BV or its main bioactive substances (e.g., melittin and bvPLA2) have been gradually studied in clinical diseases, such as osteoarthritis, musculoskeletal pain, and neuropathic pain [7,8,12]. Our earlier publications have reported that BV acupuncture (BVA) or administration of BV sub-components at ST36 (Zusanli) acupoint provided broad analgesia in oxaliplatin- or paclitaxel-induced neuropathic pain models, by selectively activating the spinal adrenergic receptors [13,14,15]. The descending noradrenergic system, which mainly originates in the locus coeruleus (LC, or A6), is the principal origin of endogenous pain modulation [16,17]. However, the effectiveness of BVA therapy and its noradrenergic mechanism (at the LC or spinal level) in the management of vincristine-induced peripheral neuropathy remains elusive.

Here, we first elucidated whether acupoint treatments with BV and its bioactive constituents (melittin or bvPLA2) could alleviate vincristine-induced behavioral hypersensitivity in rats. Second, using in vivo extracellular recording, we sought to explore the suppressive properties of BVA on the hyperexcitation of spinal wide dynamic range (WDR) neurons in rats with neuropathy. Finally, with the silencing of LC or blockade of the spinal adrenergic receptors, we investigated the specific recruitment of noradrenergic inhibitory control in BVA-induced analgesia.

## 2. Results

### 2.1. Cold and Mechanical Hypersensitivity Following Vincristine Administrations in Rats

**Figure 1:**
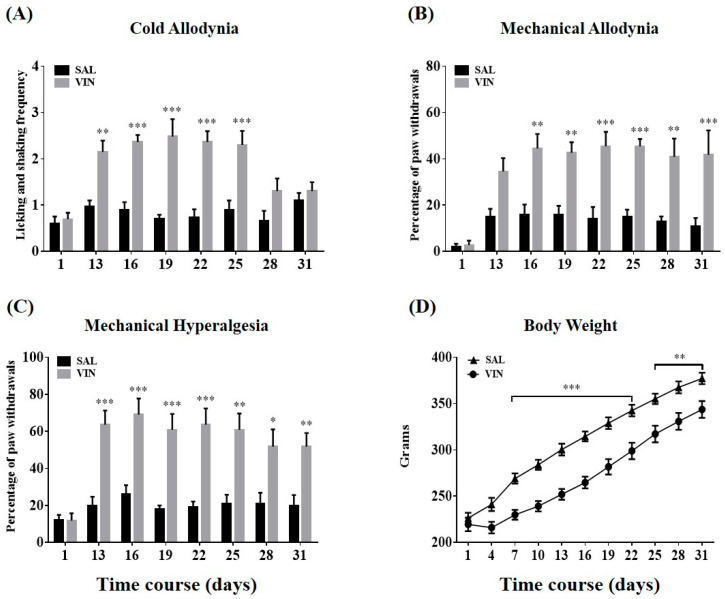
Change of behavioral hypersensitivity and body weight over time in the vincristine and control groups. Animals were intraperitoneally dosed with saline (SAL, control, *n* = 10) or vincristine (VIN, *n* = 11) once-daily for two 5-day cycles (days 1–5 and days 8–12). Rats were subjected to the behavioral evaluation just before the first dose on day 1 and between days 13 to 31, respectively (timeline: days 1, 13, 16, 19, 22, 25, 28, and 31). The acetone-elicited responses were counted over 30 s post-application (**A**). Panels (**B**) and (**C**) illustrate the responses obtained from the VFH assays (4 or 15 g filament) that were expressed as % values: (number withdrawals × 100) / (total number of tests). The linear plot represents the mean body weight (**D**). Data are expressed as mean ± S.E.M.; * *p* < 0.05, ** *p* < 0.01, *** *p* < 0.001, vs. control; by Bonferroni post-hoc test after two-way analysis of variance (ANOVA).

Ten intraperitoneal infusions of vincristine (0.1 mg/kg/day, i.p.; days 1–5 and days 8–12) induced cold and mechanical hypersensitivity in rats during 31 days of experiments. No difference between groups was observed in the baseline values, regardless of the sub-modality of assays (cold or mechanical stimulation). The licking and shaking frequency of the hind paw in response to topical acetone application (10 μL) increased in the vincristine-treated group compared to the control group (*p* < 0.01, day 13; *p* < 0.001, days 16 to 25, Figure 1A). We interpreted these aberrant symptoms as cold allodynia. For the von Frey hair (VFH) assay, mechanical allodynia and hyperalgesia (determined using 4 g and 15 g filament, respectively) were persistently observed from days 16 to 31 (allodynia, *p* < 0.01, days 16, 19, and 28; *p* < 0.001, days 22, 25, and 31, Figure 1B) or days 13 to 31 (hyperalgesia, *p* < 0.05, day 28; *p* < 0.01, days 25 and 31; *p* < 0.001, days 13 to 22, Figure 1C) after the first injection, respectively. Thus, the following evaluations were conducted during days 16 to 25, when all three types of hypersensitivity were significant. In addition, the rats subjected to vincristine administrations displayed a significantly lower increment of weight compared to the controls (*p* < 0.001, days 7 to 22; *p* < 0.01, days 25 to 31, Figure 1D).

### 2.2. Comparison of Alleviative Effects of BVA, BvPLA2, and Melittin on Vincristine-Induced Behavioral Hypersensitivity

**Figure 2:**
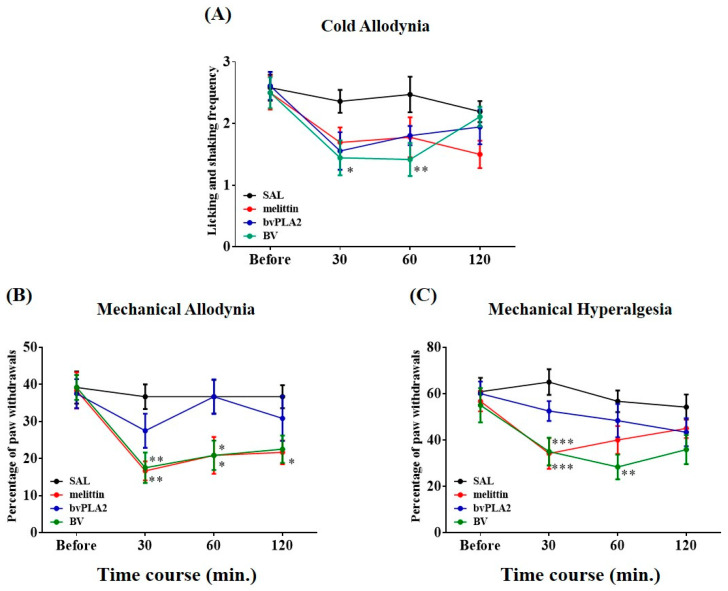
Time course of the ameliorative effects of BV, melittin, or bvPLA2 pharmacopuncture against vincristine-induced behavioral hypersensitivity. 1mg/kg of BV, 0.5 mg/kg of melittin, 0.12 mg/kg of bvPLA2, or SAL (50 μL, control) was subcutaneously dosed at ST36 (*n* = 12/group). Cold and mechanical responses (**A**–**C**) were measured four times; before application and re-examined at 30, 60, and 120 min post-dosing, respectively (timeline: Before, 30, 60, and 120). Data are expressed as mean ± S.E.M.; * *p* < 0.05, ** *p* < 0.01, *** *p* < 0.001, vs. control; by Bonferroni post-hoc test after two-way analysis of variance (ANOVA).

To compare the suppressive effects of several BV maneuvers, 48 rats with cold and mechanical allodynia and mechanical hyperalgesia were arbitrarily allocated into four groups: BV (1 mg/kg), melittin (0.5 mg/kg), bvPLA2 (0.12 mg/kg), or saline (control) was subcutaneously administrated at the ST36 acupoint. Behavioral tests were performed from the ipsilateral hind paw before acupoint injection and re-examined at 30, 60, and 120 min after application. Among these, BVA markedly attenuated the deterioration of symptoms on the acetone test, which lasted up to 60 min (*p* < 0.05, 30 min, and *p* < 0.01, 60 min, Figure 2A). In the VFH assay, the aberrant response of withdrawals to 4 g and 15 g filaments was significantly decreased after BVA, lasting for 60 min (allodynia, *p* < 0.01, 30 min and *p* < 0.05, 60 min; hyperalgesia, *p* < 0.001, 30 min and *p* < 0.01, 60 min, Figure 2B,C). Melittin treatment mitigated mechanical allodynia and hyperalgesia, as shown by obvious decrements in withdrawal responses in both the VFH assays (allodynia, *p* < 0.01, 30 min and *p* < 0.05, 60 and 120 min; hyperalgesia, *p* < 0.001, 30 min), while the behavioral change in the acetone drop test was not significant. Independent of the form of the tests, the effect of bvPLA2 was insignificant at any time point (*p* > 0.05). Based on these findings, using in vivo electrophysiological and neuropharmacological techniques, we further elucidated the analgesic mechanism of BVA, which was efficacious for both cold and mechanical complications. 

### 2.3. Vincristine Augmented Cold and Mechanical Sensory Responses of Spinal WDR Neuron in Rats

**Figure 3:**
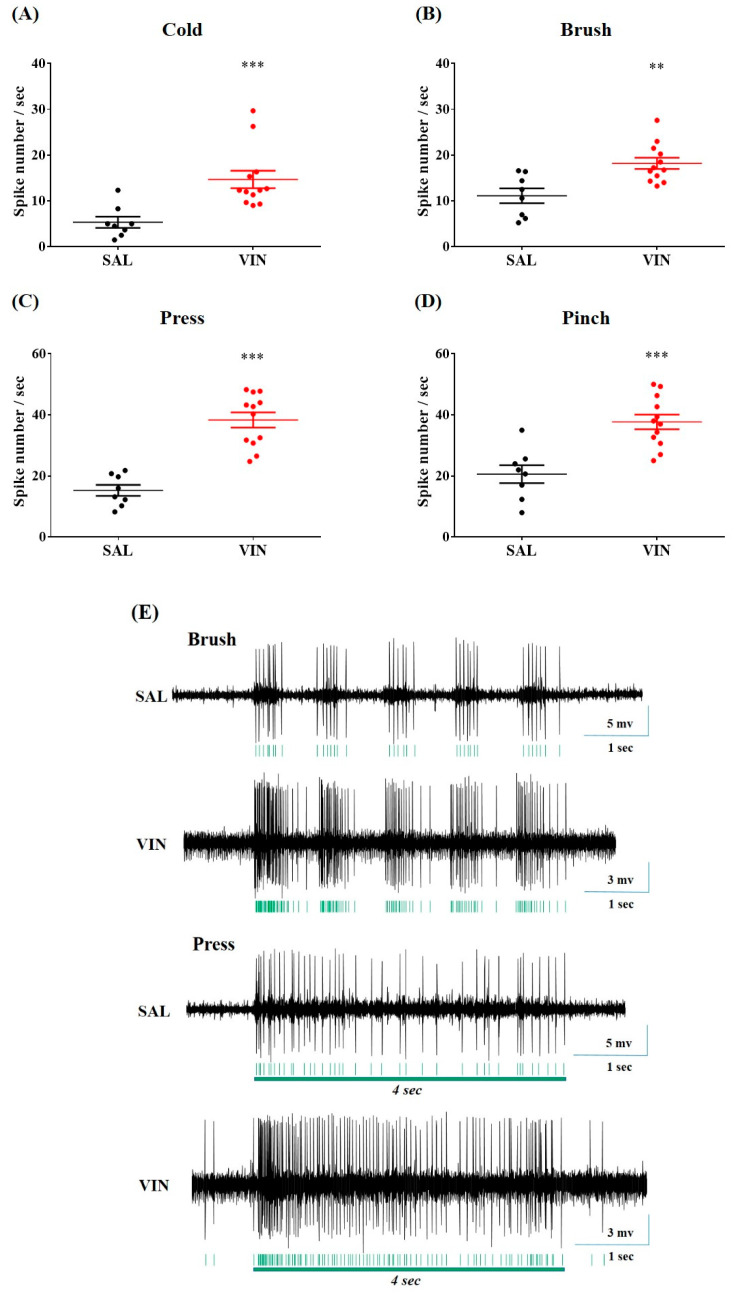
The hyperexcitation of spinal WDR neurons in rats with vincristine-induced painful disturbance. Acetone cooling, mechanical brush, press, or pinch stimuli (**A**–**D**) were applied to the peripheral receptive field of SAL (control, *n* = 8) or VIN (*n* = 12)-administered rats, respectively. The representative raw trace from in vivo recordings of WDR neurons illustrates responses to brush or press stimuli (**E**). Please note that each extracellular recording was obtained per animal. Data are expressed as mean ± S.E.M.; ** *p* < 0.01, *** *p* < 0.001; by unpaired *t*-test.

Having established different forms of behavioral hypersensitivity (Figure 1A–C), we explored the responses of spinal WDR neurons to cutaneous cold and mechanical stimuli using an in vivo extracellular recording approach (see Methods for details). Stimulus-evoked signals from 12 rats with pain behaviors, and eight control rats were analyzed (Figure 3E). In the neuropathy group, the discharge rate induced by acetone drop was significantly higher than that in the control rats (14.69 ± 1.91 vs. 5.35 ± 1.23 spikes/s, *p* < 0.001, Figure 3A). Likewise, mechanical dynamic brush, press, or pinch-evoked firing events in rats with neuropathy were markedly higher than in the controls. (brush, 18.20 ± 1.22 vs. 11.12 ± 1.62 spikes/s, *p* < 0.01; press, 38.33 ± 2.48 vs. 15.28 ± 1.80 spikes/s, *p* < 0.001; pinch, 37.69 ± 2.38 vs. 20.58 ± 2.95 spikes/s, *p* < 0.001, Figure 3B–D). These increments of stimulus-evoked activity of WDR neurons were correlated with the estimated behavioral results (Figure 1A–C), indicating the existence of CIPN generated by vincristine.

### 2.4. BVA Ameliorated Hyperexcitation of Spinal WDR Neuron Following Vincristine Administrations

**Figure 4:**
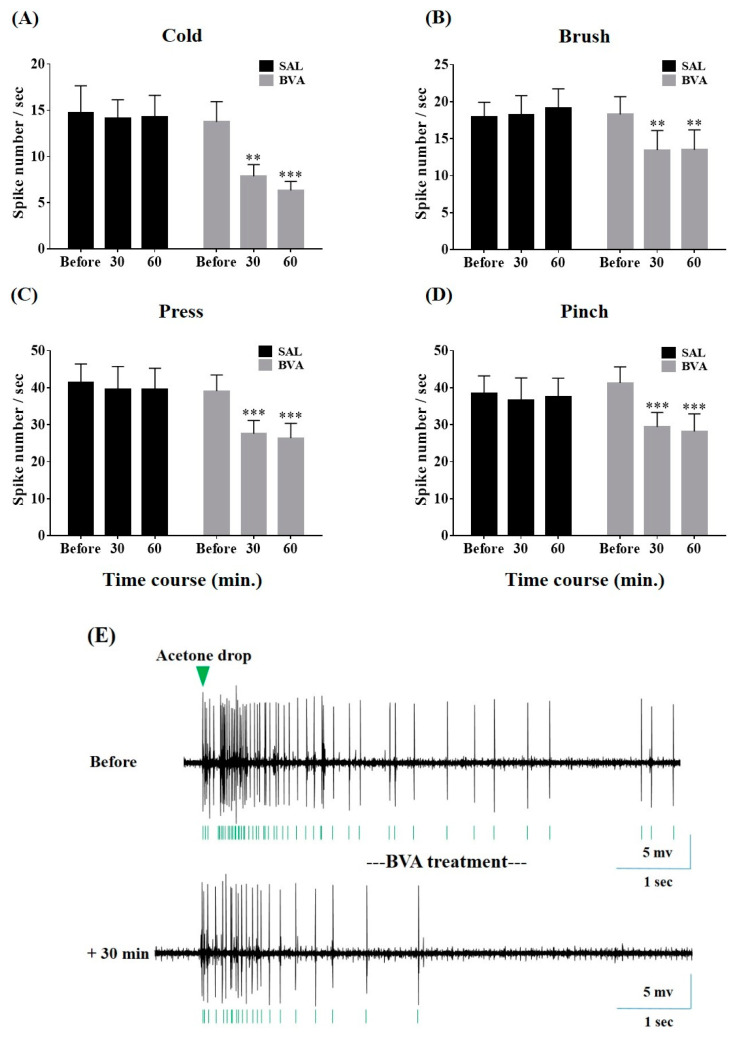
Suppressive actions of BVA on the hyperexcitation of spinal WDR neurons elicited by vincristine treatments. The rate of evoked neuronal discharge to cutaneous cold, brush, press, or pinch stimuli was obtained before, 30, and 60 min after BVA (1 mg/kg, ST36) or SAL (control) application, respectively (timeline: Before, 30, and 60; *n* = 10/group, **A**–**D**). Representative waveforms traces from extracellular recordings of WDR neurons illustrate acetone stimuli-induced firings decreased by BVA treatment (**E**). Please note that one single-unit recording was obtained per rat. Data are expressed as mean ± S.E.M.; ** *p* < 0.01, *** *p* < 0.001, vs. Before; by Bonferroni post-hoc test after two-way analysis of variance (ANOVA).

To evaluate whether BVA could inhibit neuronal hyperexcitation after vincristine infusions, in vivo extracellular recordings of spinal WDR neurons were made from rats accompanied by behavioral hypersensitivity. Once a single-unit activity was detected, ipsilateral cutaneous stimuli (cold: acetone; mechanical: brush, press, and pinch, Figure 4A–E) were applied before and at 30-min intervals for 1 h after BVA (1.0 mg/kg, ST36) or SAL treatment. Compared to the evoked single-unit activity recorded before BVA treatment, and independently of the type of stimuli, apparent decrements of the evoked firing rate were shown at 30 and 60 min after BVA (Figure 4A–D). Conversely, the neuronal responses were insignificantly changed in SAL-treated rats (*p* > 0.05). In accordance with the behavioral results mentioned above (Figure 2), our in vivo recording data demonstrated that 1.0 mg/kg of BVA at the ST36 acupoint exerted adequate analgesic effects on peripheral neuropathy elicited by vincristine.

### 2.5. Antagonism of Spinal α-Adrenergic Receptor Abolished BVA-Induced Analgesia

**Figure 5:**
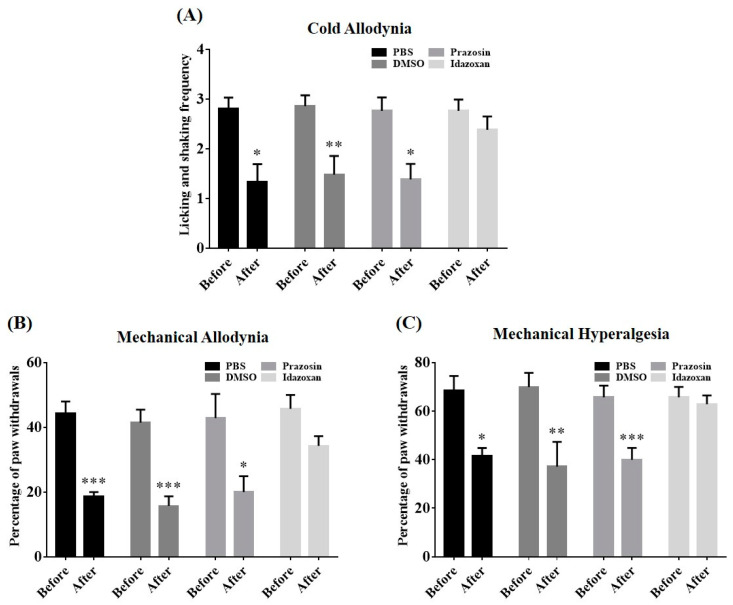
Effects of antagonism of spinal α-adrenergic receptors on the BVA-induced analgesia. Rats with neuropathy were randomly allocated into four groups (*n* = 7/group); PBS, DMSO, prazosin, or idazoxan was intrathecally injected 20 min prior to BVA (1 mg/kg, ST36), respectively. Behavioral sensitivities were assessed before the i.t. injection (Before) and 30 min after BVA (After, **A**–**C**). Data are expressed as mean ± S.E.M.; * *p* < 0.05, ** *p* < 0.01, *** *p* < 0.001; by paired *t*-test.

To clarify the alleviative effects of BVA under the specific pharmacological blockade of spinal α-adrenergic receptors (AR), the animals received the α_1_-AR antagonist prazosin (30 μg)_,_ the α_2_-AR antagonist idazoxan (50 μg), or an appropriate vehicle intrathecally 20 min before BVA application. We measured the level of neuropathy twice, just before the intrathecal (i.t.) administration and 30 min after BVA. Results revealed that pre-administration with idazoxan, not prazosin or vehicles (20% DMSO or PBS), abolished BVA-induced analgesia (Figure 5A–C). Overall, the spinal α_2_-AR, but not the α_1_-AR subtype mechanism, provided a vital contribution to the analgesic actions of BVA on cold and mechanical hypersensitivity induced by vincristine.

### 2.6. Local Blockade of the LC Reversed BVA-Induced Analgesia

**Figure 6:**
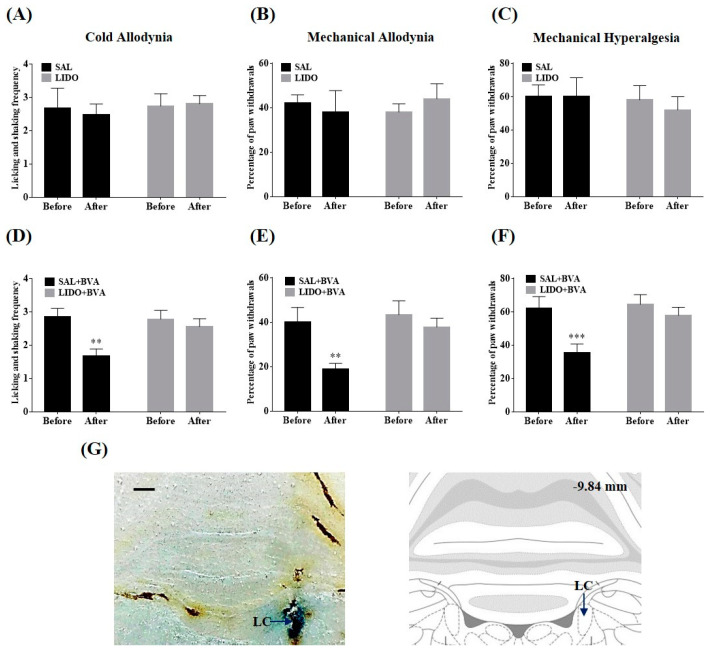
Effects of LC silencing on the BVA-induced analgesia. After cannula implantations, the rats with neuropathic signs underwent microinjection of lidocaine (LIDO, 0.5 μL, 2%) or SAL (control) into LC, respectively (*n* = 5/group in **A**–**C**, *n* = 9/group in **D**–**F**). BVA (1 mg/kg) at ipsilateral ST36 was performed immediately after microinjection (**D**–**F**). Behavioral evaluations were conducted twice; prior to microinjection (Before) and 30 min after microinjection (**A**–**C**) or BVA application (**D**–**F**), respectively. Confirmation of the microinjection position (scale bar, 500 μm; AP, ‒9.84 mm from the bregma; Paxinos and Watson, 2006; (**G**). Data are expressed as mean ± S.E.M.; ** *p* < 0.01, *** *p* < 0.001; by paired *t*-test.

To further investigate the potential participation of LC noradrenergic nuclei in the alleviative actions of BVA, local anesthesia in the LC was performed. The LC ipsilateral to the BVA injection and the behavioral test was targeted (see Methods for details). Silencing the LC region by microinjecting with lidocaine (0.5 μL, 2%) did not change the levels of cold and mechanical sensitivity in rats with neuropathy (*p* > 0.05, Figure 6A–C). In a subsequent trial, the animals received BVA treatment immediately after LC microinjection. Any aberrant symptoms were attenuated by BVA in the group that received a pre-microinjection with saline into the LC (SAL + BVA group; *p* < 0.01, *p* < 0.001, Figure 6D-F). In contrast, the ameliorative effects of BVA were completely abolished by prior microinjection with lidocaine into the LC region (LIDO + BVA group; *p* > 0.05, Figure 6D,F). Behavioral tests were performed 30 min after the BVA application, and only the results from the trials in which the microinjection was correctly into the LC area were analyzed (Figure 6G). Taken together, these results indicate the critical involvement of LC noradrenergic neurons in the suppressive actions of BVA on vincristine-induced peripheral neuropathy.

## 3. Discussion

In oncological care, the anti-cancer regimen of vincristine often results in the development of CIPN, which seriously impairs the prognosis of a survivor, who is already suffering huge stress with the existence of malignancy [6]. Fundamental painkillers (e.g., NSAIDs and opioids) have limited effectiveness on this intractable neurotoxic complication, making it more challenging to the patient [5]. As such, a novel therapeutic maneuver with reliable effects for CIPN is urgently required. Recently, our team reported that BVA, as one of the traditional mainstay methods for pain relief in East Asia, exerted remarkable effects in rodent models of peripheral neuropathy caused by taxanes or platinum-based anti-tumor substances [13,14,15,18]. Besides, the location specificity (acupoint vs. non-acupoint) of BV-induced analgesia has been determined in CIPN models [15]. In the current article, we demonstrate for the first time the ameliorative effect and mechanism of this pharmacopuncture therapy in vincristine-induced neuropathic signs in rats. 

Once-daily injection with vincristine (0.1 mg/kg, i.p.) for two 5-day cycles elicited noticeable cold and mechanical hypersensitivity in hind paws (Figure 1), which was consistent with other studies [19,20,21]. Major biological elements from BV have been shown to be efficacious for different painful deficits [7,13,14,15,22,23,24]. Among the constituents, melittin (peptides) and bvPLA2 (enzymes) are the main bioactive ingredients of BV, representing 50% and 12% in dry weight, respectively [13,25]. We compared the effect of stimulating the ST36 acupoint with melittin, bvPLA2, or BV. The ST36 is located below the knee joint [26], and moxibustion, laser, or electrical stimulation applied to this acupoint could relieve numerous symptoms of pain [27,28,29]. We first confirmed that 1.0 mg/kg BVA was efficacious for all sub-types of hypersensitivity (Figure 2). However, 0.12 mg/kg of bvPLA2 was unable to eliminate cold and mechanical abnormalities, and 0.5 mg/kg of melittin only alleviated two kinds of mechanical disturbance. For this reason, BVA treatment was chosen as a method of intervention in subsequent electrophysiological and neuropharmacological assays.

In electrophysiological analyses, we focused on the cutaneous stimulation-evoked responsiveness of WDR neurons distributed in the spinal dorsal horn, as a convergence of inputs from the somatic source could be widely relayed to those multi-receptive projection neurons [30,31,32]. Our investigation is the first to describe the alteration of peripheral cold stimuli-generated discharges arising in WDR neurons after vincristine treatments. We observed that both innocuous stimuli (acetone cooling and mechanical brush) and more noxious mechanical stimuli (press and pinch) elicited a more massive neuronal discharge under conditions of neuropathy (Figure 3). Furthermore, similar to the results of behavioral analyses (Figure 2), 1.0 mg/kg of BVA considerably protected rats from the neuronal hyperexcitation induced by vincristine (Figure 4). Growing evidence implies that the enhanced firing events of WDR neurons represent a state of pain [21,33,34], and therapy-induced decrement of these discharge rates means analgesia [35,36,37,38], indicating that our results could be promising therapeutic options for the clinical management of CIPN. 

Our next objective was to explore the functional role of the descending noradrenergic circuitry in BVA-induced analgesia. Peripheral mechanical or thermal stimulations could broadly activate the nuclei of the ipsilateral LC [39,40,41] and descending axons from the LC release noradrenaline, mostly in the ipsilateral spinal area [42,43]. By intrathecally administering α-AR antagonists or directly exposing the ipsilateral LC to lidocaine (Figure 5 and Figure 6), we determined that the BVA alleviated vincristine-induced neuropathy via activation of the spinal α_2_-AR, and the noradrenergic nuclei of the LC were involved in this analgesia. Similarly, applying acupuncture at the ipsilateral ST38 (Tiaokou) acupoint has been shown to relieve chronic shoulder pain by regulating activities of the brainstem and thalamus area [44]. α_2_-ARs are expressed in dorsal horn neurons and central terminals of the primary afferent fibers, and the activation of these receptors could attenuate the release of neurotransmitters (e.g., glutamate, substance P), thereby reducing pain transmission, and leading to considerable analgesia [16,45]. Indeed, our earlier practice has proved that oxaliplatin-induced hyperexcitation of spinal WDR neurons was suppressed by the spinal administration of adrenergic agonists [46]. Microinjections of lidocaine into the LC often induced negligible changes in pain levels [47,48,49], which was in agreement with our results (Figure 6A–C), while a recent article has revealed somewhat contrary outcomes [50]. We speculated that differences in paradigms of behavioral tests and models of pain could partially explain this discrepancy (values of overall percentage vs. up-down method in VFH tests and rodent models of CIPN vs. sural nerve injury). 

A few pre-clinical investigations have suggested that the biological components of BV effectively inhibit metastasis of hepatocellular carcinoma and proliferation of leukemic, gastrointestinal cancer, and tumor-infiltrating immune cells [51,52,53,54]. Although often unconsidered, these may be of very significance, as ideal analgesia should be appropriately efficacious against unwanted CIPN without affecting the effectiveness of anti-cancer therapy [5]. In this context, further practice is required to decipher whether antineoplastics combined with BVA could achieve better life-prolonging outcomes in the clinic.

## 4. Conclusions

In summary, we found that 1.0 mg/kg of BVA at ST36 could alleviate cold and mechanical hypersensitivity in a rat model of vincristine-induced neuropathy. This acupuncture maneuver also attenuated the hyperexcitation of spinal WDR neurons in rats with neuropathy. Moreover, BVA-induced analgesia was mediated, at least in part, by the descending noradrenergic pathway, which mainly originates from the LC. These findings suggest that BVA could be a novel analgesic candidate for vincristine-induced peripheral neuropathy.

## 5. Materials and Methods 

### 5.1. Animal

Male Sprague-Dawley (SD) rats (7 weeks old, 190–210 g) were purchased (Daehan Biolink, Chungbuk, Korea) and housed three per cage on sawdust bedding with unrestricted access to water and chow at controlled room temperature (23 ± 2 °C). Artificial lighting was regulated on a conventional 12-h light-dark cycle (dark cycle: 7 PM to 7 AM). All animal experimental protocols were approved by the Kyung Hee University Animal Care and Use Committee (KHUASP (SE) 19-011; approved January 2019 and KHUASP (SE) 20–147; approved April 2020) and performed according to the ethical guidelines of the International Association for the Study of Pain [55].

### 5.2. Behavioral Evaluation

The animals were accustomed to the testing circumstances and familiarized with the same experimenter one week before the test. On each testing day, the rats were enclosed beneath an inverted, transparent plastic box (20 × 20 × 14 cm) atop a steel mesh floor and allowed to acclimate for 30 min before the assay [15,56]. 

To measure cold allodynia, brisk reactions of the hind paw caused by acetone stimuli were monitored [57]. Using a pipette with a length of polyethylene tubing [46], 10 μL of acetone (Reagents Chemical Ltd., Kyeonggi-Do, Korea) was applied to the plantar surface of the right hind paw three times once every 10 min. The brisk shaking and licking frequency of the hind paws were quantified for 30 s [15,56]. 

To examine mechanical allodynia and hyperalgesia, rapid withdrawals of the hind paw elicited by von Frey filament applications with a bending force of 4 g (a usually innocuous stimulus) or 15 g (a usually noxious stimulus) were assessed as described in detail elsewhere [20,58]. The von Frey hair stimulus (Linton Instrumentation, Norfolk, UK) was applied vertically to the mid-plantar surface of the right hind paw ten times at 10 s intervals. The sharp withdrawal numbers were monitored and calculated as the total percentage response [13].

### 5.3. Vincristine Administration

Chemotherapy-induced peripheral neuropathy (CIPN) was established using daily vincristine infusions (0.1 mg/kg, 0.1 mg/mL, i.p.; Sigma, St. Louis, MO, USA) on two 5-day cycles with a 2-day-off schedule (days 1–5 and days 8–12) [20,21]. The vincristine group received a total of 1.0 mg/kg anti-cancer drug, and an equivalent volume of normal saline was injected into the controls.

### 5.4. BV, BvPLA2, or Melittin Acupuncture Treatment

Bee venom (BV, 1.0 mg/kg; Jayeonsaeng TJ, Kyeonggi-Do, Korea), bvPLA2 (0.12 mg/kg; Sigma, St. Louis, MO, USA), or melittin (0.5 mg/kg; Sigma, St. Louis, MO, USA) was dissolved in SAL (50 μL) and injected subcutaneously at the right hind limb ST36 (Zusanli) acupoint of animals with neuropathic signs, respectively [13]. All selected doses were guided by various publications showing analgesic effects [13,14,15,18]. ST36 is located on the tibialis anterior muscle, 5 mm lateral, and distal to the anterior tibial tubercle [14,26].

### 5.5. In Vivo Extracellular Single-Unit Recording

Extracellular single-unit recordings were obtained from the spinal dorsal horn wide dynamic range (WDR) neurons [14,46]. In brief, thoracolumbar vertebral laminectomy (T13-L2) was performed under urethane anesthesia (1.5 g/kg, i.p.; Sigma, St. Louis, MO, USA) to expose the L3-L5 region of the spinal cord. To secure in a stereotaxic frame, two spinal clamps were applied to stabilize the spinal column. The exposed spinal cord was superfused with Krebs solution containing the following (in mM): 11 glucose, 25 NaHCO_3_, 117 NaCl, 3.6 KCl, 2.5 CaCl_2_, 1.2 MgCl_2_, and 1.2 NaH_2_PO_4_ saturated with O_2_/CO_2_ gas (19:1 *v*/*v*), 38 ± 1 °C at a rate of 15 mL/min [40,46]. After the removal of the dura mater, a 10-MΩ tungsten electrode (FHC, Bowdoin, USA) was smoothly inserted into the dorsal horn and manually descended using a microdrive (Narishige, Tokyo, Japan). The receptive field of the isolated WDR neuron was identified by stimulating the surface of the right hind paw in the following order: brush, press, pinch, and acetone drop [14]. Among these, a brush stimulus was given by stroking the receptive field with a camel brush five times for 4 s. Press stimulus was performed by pressing the center of the receptive field for 4 s using the blunt tip of the camel brush with a diameter of 0.5 cm and a magnitude of approximately 20 g. Each 3-s pinch stimulus was applied to the skin with toothed forceps (11022-14, Fine Science Tools, Heidelberg, Germany) [14]. For cold stimulation, 10 μL of acetone was applied to the receptive field [46]. Stimulus-evoked signals were amplified (DAM80, WPI, Sarasota, USA) and digitized (Digidata 1440A, Axon Instruments, Foster City, CA, USA), followed by offline data analysis with Spike 2 v6.0 (Cambridge Electronic Design, Cambridge, UK) [46].

### 5.6. Cannula Implantation and Drug Microinjection into the LC

Rats with cold and mechanical hypersensitivity were stabilized in a stereotaxic frame under isoflurane anesthesia (2.5–3.0%; Hana Pharm. Co., Kyeonggi-Do, Korea) delivered with O_2_/NO gas (1:1 *v*/*v*) [59]. A hole was drilled in the skull, and a stainless-steel guide cannula (26 GA, C315G; Plastics One, Roanoke, VA, USA) was lowered to 1.0 mm above the right side LC with the following coordinates: AP, -9.8 mm from the bregma; ML, 1.3 mm from the midline; DV, 6.5 mm below the skull [60]. Each cannula was placed in the skull with two screws using dental cement and plugged with a dummy cannula (C315DC/1; Plastics One, Roanoke, VA, USA) [47,59]. Following surgery, the animals underwent a 6-day period of recovery [61]. 

A microinjection was performed under isoflurane-induced (1–1.5%) light sedation [61]. The dummy was replaced with an injection cannula (33 G, C315I; Plastics One, Roanoke, VA, USA) protruding 1.0 mm beyond the end of the guide cannula (i.e., DV, 7.5 mm below the skull). 0.5 μL of SAL or 2% lidocaine was infused over 30 s via a Hamilton syringe (10 μL; Hamilton Co., Reno, NV, USA) attached to a polyethylene (PE-10) tubing to the injection cannula [47,49,61]. To minimize backflow, the injection cannula remained in place for an extra 5 min. After receiving urethane anesthesia (2.0 g/kg, i.p.), animals were microinjected with trypan blue (0.5 μL; Sigma, St. Louis, MO, USA) to mark the position. Each pons was removed, sliced into coronal sections (20 μm) [61]. We only analyzed data from the tests in which the microinjection was correctly placed into the LC region (*n* = 38 total rats, and *n* = 28 rats with correct implantation).

### 5.7. Spinal Antagonist Treatment

To clarify which spinal α-AR subtype mediates the analgesic actions of BVA in vincristine-administered rats, specific α-AR antagonists were administered intrathecally (i.t.) 20 min before BVA treatment [13,14]. The α_1_-AR antagonist prazosin (30 μg; Sigma, St. Louis, MO, USA) and α_2_-AR antagonist idazoxan (50 μg; Sigma, St. Louis, MO, USA) were dissolved in 20% DMSO (Sigma, St. Louis, MO, USA) or PBS (10 μL), respectively [13,14]. In a prone position under isoflurane anesthesia (2.0–2.5%), each animal was subjected to an i.t. administration of antagonist or vehicle via a direct lumbar puncture at the L5-L6 intervertebral level [14,62].

### 5.8. Experimental Schedule and Schematics of the Putative Central Mechanism of BVA-Induced Analgesia

**Figure 7:**
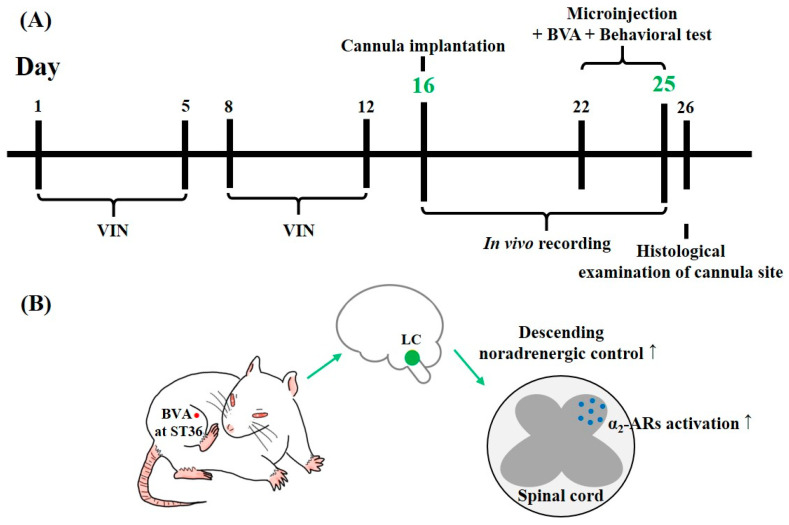
Timeline of the investigation and schematic illustration of BVA-induced analgesia. In vivo electrophysiological and neuropharmacological tests were performed during days 16 to 25, when all three forms of hypersensitivity were significant (**A**). Panel (**B**) illustrates that BVA at the ST36 acupoint activates spinal α_2_-ARs via recruiting the descending noradrenergic pathway from the LC.

Repeated i.p. infusions of vincristine (days 1–5 and days 8–12) elicited all three types of hypersensitivity at days 16 to 25. As a result, in vivo electrophysiological and neuropharmacological assays were performed in this period (Figure 7A). We speculated that BVA at the ST36 acupoint could alleviate vincristine-induced peripheral neuropathy through the specific recruitment of the central noradrenergic pathway, which mainly originates in the LC (Figure 7B). 

### 5.9. Statistics

Statistical analysis was performed using Prism v7.0 (GraphPad Software, La Jolla, CA, USA), and differences were considered significant at *p* < 0.05. Data are presented as mean ± standard error of the mean (S.E.M.). Statistical testing was performed with two-way ANOVA followed by Bonferroni’s multiple comparison test, unpaired *t*-test, or paired *t*-test as appropriate.
